# Reduction of care-relevant risks to older patients during and after acute hospital care (ReduRisk) – study protocol of a cluster randomized efficacy trial in a stepped wedge design

**DOI:** 10.1186/s12877-022-03442-4

**Published:** 2022-09-15

**Authors:** Anne Göhner, Elena Dreher, Felix Kentischer, Christoph Maurer, Erick Farin-Glattacker, Rieka von der Wart, Boris A Brühmann, Andy Maun, Vitalii Minin, Claudia Salm, Alexander Ritzi, Mario Sofroniou, Sebastian Voigt-Radloff

**Affiliations:** 1 Center for Geriatric Medicine and Gerontology Freiburg, Faculty of Medicine and Medical Center - University of Freiburg, Lehenerstrase 88, Freiburg, 79106 Germany; 2 Section of Health Care Research and Rehabilitation Research, Institute of Medical Biometry and Statistics, Faculty of Medicine and Medical Center-University of Freiburg, Hugstetterstraße 49, Freiburg, 79106 Germany; 3 Institute of General Practice/Family Medicine, Faculty of Medicine and Medical Center-University of Freiburg, Elsässerstraße 2n, Freiburg, 79110 Germany

**Keywords:** Activities of daily living, Aged, Delivery of health care, integrated, Evidence-based-practice, Geriatric assessment, Multimorbidity, Risk management, Telemedicine

## Abstract

**Background:**

Older patients are at an increased risk of hospitalization, negatively affecting their health and quality of life. Such patients also experience a lack of physical activity during their inpatient stay, as well as being at increased risk of delirium and inappropriate prescribing. These risk factors can accumulate, promoting a degree of morbidity and the development of cognitive impairment.

**Methods:**

Through the ReduRisk-program, patients at risk of functional impairment, immobility, falls, delirium or re-hospitalization shortly after hospital discharge, will be identified via risk-screening. These patients will receive an individually tailored, multicomponent and risk-adjusted prevention program. The trial will compare the effectiveness of the ReduRisk-program against usual care in a stepped-wedge-design, with quarterly cluster randomization of six university hospital departments into intervention and control groups. 612 older adults aged 70 years or more are being recruited. Patients in the intervention cluster (*n* = 357) will receive the ReduRisk-program, comprising risk-adjusted delirium management, structured mobility training and digitally supported planning of post-inpatient care, including polypharmacy management. This study will evaluate the impact of the ReduRisk-program on the primary outcomes of activities of daily living and mobility, and the secondary outcomes of delirium, cognition, falls, grip strength, health-related quality of life, potentially inappropriate prescribing, health care costs and re-hospitalizations. Assessments will be conducted at inpatient admission (t0), at discharge (t1) and at six months post-discharge (t2). In the six-month period following discharge, a health-economic evaluation will be carried out based on routine health insurance data (t3).

**Discussion:**

Despite the importance of multicomponent, risk-specific approaches to managing older patients, guidelines on their effectiveness are lacking. This trial will seek to provide evidence for the effectiveness of a multicomponent, risk-adjusted prevention program for older patients at risk of functional impairment, immobility, falls, delirium and re-hospitalization. Positive study results would support efforts to improve multicomponent prevention and the management of older patients.

**Trial registration:**

German Clinical Trials Register, DRKS00025594, date of registration: 09/08/2021.

## Background

In 2021, average life expectancy was 73 years of age worldwide and 81 years of age in the European Union and Germany [1]. About 10% of the world’s population was older than 64 years of age, with the highest proportions in Europe and the United States at 19 and 17%, respectively [2]. In Germany, every fifth person was older than 64 years of age (22%) [1]. The demographic changes seen in Germany are thus well-advanced in comparison with other nations. In the coming years, the care of older people in Germany will become a great challenge due to increased life expectancy, the lack of skilled workers and a decline in family support structures [3, 4]. Older inpatients spend 80% of their time confined to a bed, walking only 43 min per day, even if they were able to walk on admission [5]. Insufficient physical activity during an acute inpatient stay can lead to functional decline, frailty, complex transitions back into their home environment, and even death in older patients, regardless of the severity of the disease or comorbidity [5–9]. Additionally, older frail patients in intensive care units are at an increased risk of inpatient short- and long-term mortality and a reduced likelihood of being discharged home [10, 11]. Delirium is associated with increased morbidity, mortality, cognitive impairment, dementia progression and institutionalization rates [12–15]. Hospital admission increases the risk of potentially inappropriate prescribing in older patients [16, 17], particularly affecting their multimorbidity.

Multimorbidity represents a substantial burden on the older population: of the over 70-year-olds in Germany 56% have two to four chronic illnesses, with 21% having five or more [18]. Older people with multimorbidity are more frequently readmitted to hospital, being at risk of disruptions to cross-sectional care, higher health care costs, reduced autonomy, functioning and life satisfaction [19–23]. The prevalence of illnesses typical of older patients in acute inpatient care is high: 85% ADL problems, 59% mobility deficits, 61% polypharmacy, 52% malnutrition. However, these illnesses are poorly reported in physician letters (ADL and mobility problems below 50%), despite high rates of functional decline (33%) and mortality (35%) in the year following discharge [24]. In order to address these age-related problems, and aid prevention, several well-established, single-disease interventions already exist:

Strength training leads to improvements in general physical ability, walking speed, transfer from sitting to standing, and muscle strength [25]. Furthermore, the training of gait, balance, coordination or functional tasks improves performance in the Timed Up & Go test, in walking speed and the Berg Balance Scale [26]. Strength or multimodal mobility training in older people with reduced physical performance demonstrates similar positive effects on muscle strength, balance and walking speed [27]. Balance-orientated training programs, with an intensity of more than 3 h per week, reduces the frequency of falls in older people living at home [28], whilst structured training reduces fractures caused by falls [29]. Early mobilization reduces the incidence of intensive care-acquired weakness, improves functional capacity, as well as increasing the number of ventilator-free days and the rate of discharge-to-home for patients with critical conditions [30]. Training of older patients in the acute inpatient care setting has positive effects on discharge-to-home, hospital days and hospital costs [31]. More recent systematic reviews of acute inpatient care, confirm the positive effects of training programs and show potential promise [32]. However, there is heterogeneity in the definition of the intervention, its intensity, monitoring adherence and evaluation methods, with no greater effectiveness being demonstrated through individualization of the training [33].

Multi-component delirium-prevention programs are suitable for patients at risk of delirium in an acute inpatient setting [34]. Delirium can be avoided if risk factors such as infection, dehydration, and related concurrent medications [35] are observed and addressed [36]. A delirium prevention program tested in Germany (PAWEL) was able to reduce postoperative delirium occurrence and days with delirium in older patients undergoing orthopedic or abdominal surgery [37].

Care planning as part of discharge management reduces re-hospitalization rates at three-month follow-up for older patients [38]. Moreover, complex programs consisting of at least five components demonstrated a positive effect on 30-day re-hospitalization rates [39]. A recent analysis of old age specialist co-management programs strongly recommended starting co-management within 24 h of hospital admission, using clearly defined criteria for selecting appropriate patients [40]. Although in Germany, there is a pre-existing expert standard for discharge management in nursing [41], innovative and digitally supported trans-sectoral care models are needed [42]. In summary, trans-sectoral care management is a promising intervention for older, chronically ill patients [38, 43, 44].

The management of multiple medications is a difficult yet relevant task, especially in light of the fact that most patients are prescribed medications from several physicians simultaneously involved in their care. The management of inpatient polypharmacy does not appear to reduce re-hospitalization rates, but does reduce the number of contacts with emergency departments [45]. There is some evidence to suggest that medication reviews reduce the number of missed medications in the elderly, but not the number of potentially inappropriate prescriptions [46]. Current systematic reviews on the management of polypharmacy in physically frail, older patients with hypertension, diabetes mellitus, late-life depression and dementia, indicate that the evidence is still inadequate to enable practical recommendations to be made [47–49]. Since 2016, patients in Germany who take at least three prescribed medications have a legal entitlement to a medication plan, which itemizes their medication information into a standardized form. However, there are still challenges and omissions in the implementation of discharge management and medication plans [50].

eHealth solutions can be used to support outlined interventions, for instance, in delaying functional decline [51], providing evidence-based health information [52] or supporting personal health information management [53], whilst also increasing the acceptance and use of internet-based technologies [54]. Access to the internet, hardware availability and sufficient technical skills are essential for using eHealth solutions. As of 2019, only 7% of internet users worldwide were aged 65 years and above [55]. In Germany, the current proportion of people over 70 who are offline is 14%, which corresponds to approximately 9 million people [56]. Against the backdrop of the Corona crisis and the accompanying push to digitalization, easy access to equipment and e-learning opportunities for older people is needed [57]. Initial projects to close the technology gap are promising, but have struggled to reach the technically unskilled, very old or vulnerable people [56].

What is currently missing is a risk-adjusted approach, that combines differing indication-specific interventions in a practicable way [58]. The risk of functional decline in the post-discharge phase could be assessed via simple screening questions at inpatient admission [59]. The standardized assessment of risk factors and the early addressing of deficits in ADL, mobility and cognitive function has not as yet become widely established in the routine processes of acute inpatient care of older patients in Germany. The ReduRisk-program (reduction of care-relevant risks to older patients in and after acute hospital care) aims to close this gap by combining risk-specific screening at inpatient admission with a variety of evidence-based intervention modules, through a targeted, risk-orientated and thus cost-effective manner.

## Methods and design

### Study aim and objectives

Based on findings that outline effective interventions, ReduRisk combines (1) risk screening of older patients in acute inpatient care and (2) subsequent care management, with risk-adjusted (a) structured mobility training, (b) individualized delirium prevention, and (c) patient-orientated and digitally supported intervention-modules for self-training, care planning and polypharmacy management. In order to close the technology gap, the ReduRisk-program offers participants the opportunity to take part in several modules on using a tablet device, including a crash course on its use and continuous guidance during the inpatient period.

The aims of the study are:

- To assemble an evidence-based, needs-orientated intervention and optimize it under routine conditions,
- To implement the intervention at six university hospital departments,
- To evaluate the short- and medium-term effects of the intervention and its cost effectiveness in comparison to usual care,
- To make the intervention available as a new form of care, provided the effects are positive,
- and maintain the replicability of all implementation and evaluation procedures in future studies.

#### Primary hypothesis

Our primary hypothesis is that the ReduRisk-program will significantly reduce immobility and decline in activities of daily living, compared to usual care at discharge.

#### Secondary hypothesis

Compared to usual care, we also hypothesize that the ReduRisk-program will:

1. Reduce immobility and decline in activities of daily living at six months post-discharge,
2. Reduce the risk of falls, delirium and cognitive dysfunction at discharge and at six months post-discharge,
3. Reduce re-hospitalization and institutionalization rates within the six months post-discharge,
4. And reduce the overall cost of care over a six-month time-frame.

### Trial design and setting

ReduRisk is a monocentric, cluster-randomized study with a stepped-wedge design conducted at the University of Freiburg Medical Center. Project management, optimization, training, supervision and recruitment will be carried out by the Center for Geriatric Medicine und Gerontology, University of Freiburg Medical Center (ZGGF). Data collection and subsequent implementation of the intervention will be carried out by the ZGGF and the Institute of Primary Care at the University of Freiburg Medical Center (IfA). The Section of Health Care and Rehabilitation Research, University of Freiburg Medical Center (SEVERA), will deal with the data management, evaluation and health economics analysis. Health care costs and re-hospitalization will be evaluated in cooperation with the AOK (Allgemeine Ortskrankenkasse), the largest statutory health insurance organization within the University of Freiburg Medical Center region.

#### Eligibility criteria und risk screening

Target groups of the ReduRisk-program will include:

- Patients treated in one of six participating departments at the University of Freiburg Medical Center,
- Patients aged 70 years or more,
- Patients at an increased risk of functional impairment, immobility, falls, delirium or re-hospitalization,
- And patients who have given their written informed consent to participate.

Patients undergoing terminal-phase palliative care will be excluded. In order to verify those with an increased risk, risk screening will be carried out using the following parameters in both intervention and control groups:

- Risk of functional decline and re-hospitalization: “Identification of Seniors at Risk Screening Tool” (ISAR, 0 = no risk to 6 = very high risk) [60]. Patients with an ISAR-score ≥ 3 or a 'yes' for ≥ 6 medications will be considered to have an increased risk and will thus be included in the study.
- Risk of immobility, falls, frailty, re-hospitalization and mortality: “Short Physical Performance Battery” (SPPB, 12 = good to 0 = bad) [61–63]. Patients with an SPPB-score ≤ 9 will be considered as being at an increased risk and will thus be included in the study.
- Risk of delirium: as outlined in the guidelines of the University of Freiburg Medical Center on the prevention and treatment of delirium (UKF-Delirium standard) [64] and the “3D-CAM” [65]. Patients with ≥ 1 risk factor will be included in the study; those with acute delirium will however be excluded due to an inability to give informed consent.

#### Intervention group

Study participants in the intervention group will receive an individualized, risk-adjusted program consisting of up to six modules, carried out by trained study staff. These will be health professionals including physicians, nurses, and physiotherapists, termed ‘interventionists’. To avoid intervention-overload due to cumulative risks, the ReduRisk program will consist of a maximum of three thirty-minute sessions per day. In several of the intervention modules, study participants will be able to choose whether they want to complete the module using a tablet device or paper documentation, according to their preferences.

#### Modules of ReduRisk

##### Module (1) Tablet device crash course (triggered by tablet device preference in at least one intervention module)

In order to provide confidence in tablet device use, study participants who chose to complete at least one intervention module using a tablet device, will be offered a crash course by the interventionists. During the crash course, interventionists will explain the basic functions of the tablet device and the areas and functions of the ReduRisk-program. The course will last thirty to sixty minutes. Content will be adapted to an individual's previous knowledge and patients will receive a handout describing the essential functions of the tablet device.

##### Module (2) Individualized health information

To improve health and problem-solving skills, the interventionists will provide all participants in the intervention group (regardless of risk-screening) with structured, quality-assured information and planning aids, such as health information and guidelines from the AWMF (The Association of the Scientific Medical Societies in Germany), the ÄZQ (The German Agency for Quality in Medicine), the IQWIG (The Institute for Quality and Efficiency in Health Care) and communication guidelines for consultations with a physician based on previous studies (e.g. [52, 66–69]). Information needs and post-discharge care needs will be identified using a tablet device or paper folder, via a self-developed, structured assessment of the following subject areas: personal hygiene, well-being, exercise, emergency preparedness and household management.

##### Module (3) Care planning with individualized health information (triggered by the cut-off value ISAR ≥ 3 or a "yes" to ≥ 6 medications [60])

If this module is triggered, participants will move from module 2 to 3, and the results of the information-needs and care-problem analyses will then serve as a starting point for the preparation of an individualized care plan. Interventionists will accompany study participants through a stepwise approach in the identification and definition of individual care problems, care goals and resulting solutions. During care planning, further information needs will be queried and the necessary information provided. In the case of polypharmacy, the care plan will focus on the reliable maintenance of a complete and up-to-date medication list. The tablet devices contain a framework for care planning. If a participant would like to plan their care without the use of a tablet device, a paper folder containing structured forms will be provided.

##### *Module (4) Mobility training (triggered by cut-off value: ISAR* ≥ *3 or SPPB* ≤ *9 *[61–63]*)*

An effective, tried and tested mobility training tool from Martínez-Velilla [70] has been adapted for our purposes, which includes strength, balance, flexibility, and cardiovascular training with walking exercises [25–27, 32, 71]. We have produced training videos for the participants with different levels of difficulty for each exercise. Patients have been assigned to one of the following risk-adjusted exercise groups: (1) “bed-mobile”, with strength, flexibility and cardiovascular training in bed (SPPB 0–3); (2) "room-mobile", with strength, balance, flexibility and cardiovascular training with walking exercises with holding, weight relief, personal support (SPPB 4–6) or (3) "ward-mobile", with strength, balance, flexibility and cardiovascular training with walking exercises outside the ward room (SPPB 7–12). Each session consists of 10 min preparation time, including motivation and safety-concerns, with 20 min allocated to training. The intensity can be increased and decreased as necessary. Participants receive training as video-based instructions on a tablet device or as printed instructions with a training diary. Training will initially be carried out under the supervision of the interventionists, thereafter supported by trained assistants and, as long as it is safe to do so, further developed into self-training.

##### Module (5) Delirium prevention (triggered by one or more predisposing risk factors according to risk screening by UKF-delirium standard [64])

The intervention modules of the Aktiver® Delirium Prevention Program, a part of the PAWEL program [37], have been integrated into the ReduRisk-program. During the first intervention contact, a personalized delirium risk profile will be drawn up, with appropriate preventative measures planned and implemented by the interventionists during the hospital stay. The delirium prevention module will be undertaken by the interventionists and supported by assistants, a delirium prevention team (Aktiver) and ward staff of the University of Freiburg Medical Center. The Aktiver team will support the interventionists by implementing measures for delirium prevention in the areas of orientation, activation, mobilization, sleep promotion and relaxation, diagnostic and meal support. In order to be able to react to changes in the cognitive status, the interventionists will be responsible for monitoring preventative measures. Delirium screening using the Delirium Observation Screening Scale (DOS) [72] will be performed by the ward staff of the supervising department at every shift. The results will be monitored by the interventionists. Positive Delirium screening results will be verified using the 3-Minute-Diagnostic-Confusion Assessment Method (3D-CAM)[65]. If delirium is identified, this will be recorded in the electronic patient file of the department and, if possible, communicated in person to the ward staff. In addition, the interventionists will conduct a daily chart review to identify evidence of delirium and other changes in cognitive status. If necessary, the delirium prevention measures can be adjusted accordingly. Delirium prevention will end with the inpatient intervention phase.

##### Module (6) Polypharmacy management (triggered by the cut-off value ISAR "yes" to ≥ 6 medications [60])

A family physician, the study doctor, will conduct the polypharmacy management module. During the inpatient intervention phase, the study doctor will prepare a medication recommendation for the ward doctors responsible for the patient’s management, based on an analysis of the hospital documentation and a face-to-face conversation between the study doctor and the participant. Recommendations for medication changes, in order to detect Potentially Inappropriate Medications (PIMs) and Potential Prescribing Omissions (PPOs), will be based on the STOPP/START criteria version 2 [73] and the medication appropriate index [74], in addition to the professional experience of the study doctor involved. Further enquiries by the attending physicians regarding these recommendations will be possible during the course of inpatient treatment. If necessary, this can lead to an adjustment of the recommendations. Aspects of medication and drug safety will be brought to the attention of patients and their carers, as well as the managing physicians, through specific handouts. Upon discharge, study participants and their family physicians will receive a letter with the contact details of the study doctor and an offer of an outpatient medication review within the study period.

#### Intervention phases

During the inpatient phase, the study participants will receive the ReduRisk-program with its combined modules up to three times a day for thirty minutes, with a face-to-face consultation prior to discharge, in which the need for further support will be evaluated.

**Fig. 1:**
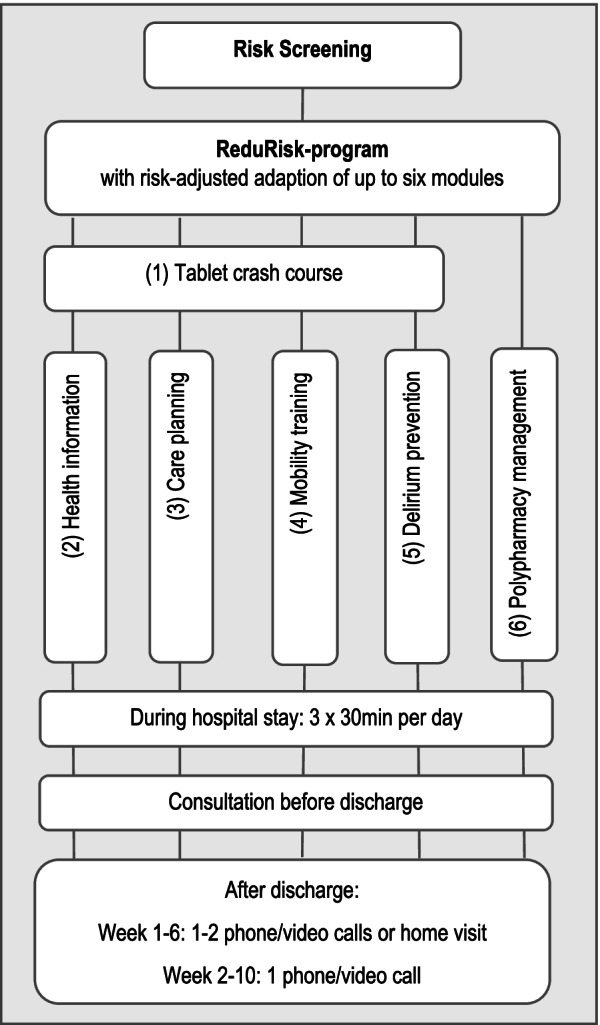
ReduRisk-program

In the post-discharge phase, one or two needs-specific contacts will take place, with the first being conducted approximately 1–6 weeks following discharge, as a video or phone call, or as a home visit if required. The focus therein will be on individual successes or problems with the ReduRisk-program. If necessary, adaptations will be made, further contents provided, and study participants and their relatives will be supported in their documentation and exercises. Approximately 2–10 weeks following discharge, a final telephone contact will take place. The focus will then be on how study participants and their relatives could address or solve current and possible future health and care problems with the family physician and, if necessary, offered counselling and care facilities in their region. Subsequently, study participants will independently follow the individualized modules of the ReduRisk-program until the end of the intervention phase six months post-discharge. Intervention phases and program modules are shown in Fig. 1 “ReduRisk-program”.

#### Control group

Study participants in the control group will receive the usual care.

#### Test of practicability and quality maintenance

Prior to the start of the study, a first draft of the study information was presented, along with consent forms, program contents, study materials and the tablet-based care plan to pilot-patients. These were optimized according to patient feedback. Additionally, the practicability of the multicomponent interventions and the study procedures were tested and optimized prior to the start of the intervention period. At the start of data collection, study staff and staff in participating departments were trained on the study contents and procedures. The inpatient and post-discharge contacts were quality-assured through manuals, checklists and conversation guidelines.

#### Criteria for modification and discontinuation

If adverse events occur or new needs arise during the intervention phase, these will be documented and the intervention adjusted if necessary. The criteria for dropout are: (1) the participants or legal representatives withdraw informed consent, (2) death of the participant, (3) lack of availability, (4) moving out of the catchment area of the University of Freiburg Medical Center (more than 100 km away), (5) the patient is not yet sufficiently familiar with the ReduRisk-program and has been transferred to a department that does not belong to an intervention cluster.

### Outcome measures and assessments

#### Primary and secondary outcomes

The following primary outcome measures are the patient-related endpoints:

- Mobility, assessed by SPPB [75],
- Activities of daily living, assessed by Barthel Index [76].
  The following secondary outcome measures are the patient-related endpoints:
- Delirium, assessed by 3D-CAM (at t2 by retrospective survey) [65],
- Cognition, assessed by Montreal Cognitive Assessment (MOCA) [77],
- Falls, assessed by number and injuries / fractures,
- Grip strength, assessed by dynamometer,
- Health-related quality of life, assessed by SF-12 [78],
- Potentially Inappropriate Medications (PIM), assessed by STOP / START criteria, version 2 [73].
- Health economics: health care costs and re-hospitalization from AOK claims data (Allgemeine Ortskrankenkasse).

#### Assessment for evaluation

Blinded and trained assessors will evaluate all study participants at three consecutive time points: at admission (t0), discharge (t1) and at six months post-discharge (t2). For the health economic evaluation (t3), costs and re-hospitalizations will be assessed based on claims data from the statutory health insurance organization the AOK. The measurement concept including measurement time points and assessment instruments used to measure primary and secondary outcomes is shown in Table 1 “Assessment and time of measurement”.

**Table 1:** Assessments and time of measurement

Month	0	At discharge	6	18
**Time of measurement**	**t0**	**t1**	**t2**	**t3**
**Risk-Screening**				
Functional decline and re-hospitalization (ISAR)	X			
Falls/ Immobility (SPPB)	X			
Delirium (risk-screening according to UKF-delirium standard, 3D-CAM)	X			
**Basic Data**				
Sociodemographic data	X			
Diagnoses	X			
**Primary Outcome**				
Mobility (SPPB)	X	X	X	
Activities of daily living (Barthel index)	X	X	X	
**Secondary Outcome**				
Cognition (MOCA)	X	X	X	
Delirium (3D-CAM)	X	X	X	
Falls (Number in total, injuries / fractures)	X	X	X	
Grip strength (Dynamometer)	X	X	X	
Health-related quality of life (SF-12)	X	X	X	
Potentially inappropriate medications (STOPP/START)	X	X	X	
Re-hospitalization (AOK—claims data)		→		X
Health care costs (AOK—claims data)	→			X

*3D-CAM *Confusion Assessment Method, *AOK *Allgemeine Ortskrankenkasse (Statutory Health Insurance), *ISAR *Identification of Seniors At Risk, *MOCA *Montreal Cognitive Assessment, *SPPB *Short Physical Performance Battery, *STOPP/START *Screening Tool of Older Persons’s Prescriptions / Screening Tool to Alert to Right Treatment, *UKF-Delirium Standard *Internal Standard for Delirium Prevention and Treatment of the University of Freiburg Medical Center

#### Sample size and power calculation

**Fig. 2:**
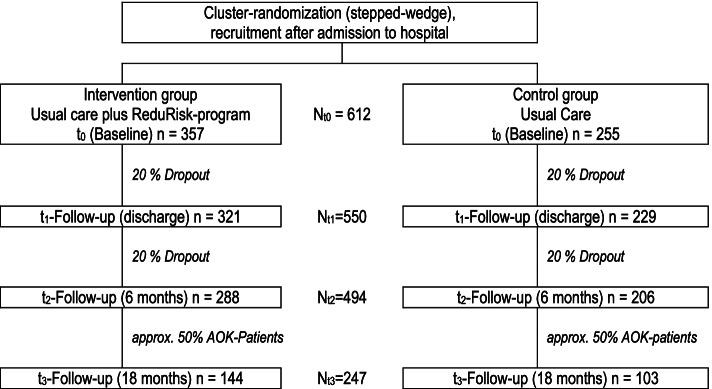
Trial design

We aim to recruit 612 patients to the study, with 357 patients in the intervention group and 255 in the control group. Assuming a dropout rate of 20%, the number of study participants in the intervention and control group should correspond to *N* = 550 at t1 and *N* = 494 at t2. For the health economic evaluation, the number of cases *N* = 247 will be assumed, as approximately 50% of all participants are AOK patients. The planned patient flow is shown in Fig. 2 “Trial design”.

#### Process evaluation

Contextual factors are essential for the evaluation and sustainable implementation of complex interventions [79]. For this purpose, we will assess (1) contextual factors at the patient level (sociodemographic background, diagnoses) through assessment of routine clinical data, (2) patient adherence to the intervention through structured interviews during the inpatient and post-discharge phases and surveys of tablet device use and (3) adherence of the interventionists to the manual through the documentation of the accomplished interventions. Additionally, we will address the following topics at the organizational level through seven semi-structured, 45-min focus group interviews with study staff and study-involved staff from the six departments: a) Feasibility of the intervention modules, b) Obstacles and factors conducive to implementation in accordance with the manual, c) Perceived acceptance of the study participants, d) Benefit and suggestions for optimization.

#### Recruitment of participants

Interventionists will compile a daily list of newly admitted patients aged 70 and over hospitalized at one of the six participating departments of the University of Freiburg Medical Center. Patient records will be checked for the presence of inclusion criteria. The interventionists will then introduce the ReduRisk-project to potentially eligible patients through the use of comprehensive study information, and informed written consent from those interested in participating. If patients are incapable of giving consent due to severe cognitive impairment, informed written consent for study participation can be obtained from the legal guardian. In this case, a proxy assessment will be conducted with the legal guardian or with the relatives of the person concerned. If patients subsequently show an increased risk of functional impairment, immobility, falls, delirium or re-hospitalization during risk-screening, they can still participate in the study.

#### Assignment and randomization

ReduRisk is a monocentric stepped-wedge study with quarterly, computer-generated, cluster randomization of six departments to intervention and control groups (1 hospital department = 1 cluster). The study center will carry out allocation assignments independently of ward staff and their respective interventionists.

#### Blinding

Due to the intervention and randomization design, it will not be possible to blind the study participants and the interventionists, who are responsible for recruiting and carrying out the intervention, or to assign them to the intervention arm in a hidden manner. In order to prevent any influence on the assessment, blinded and trained assessors will carry out the assessment survey both in the intervention and control groups. Study participants and interventionists are required not to reveal the allocation status. Due to pseudonymization, the outcomes will be analyzed without knowledge of the study arm allocation, apart from the process evaluation.

### Statistical analyses

The superiority of the intervention group over the control group with regards to the two primary endpoints, mobility and ADL function, will be tested as the hypothesis. Medium effect sizes are expected to be in keeping with Martínez-Velilla et al. [70] (Assuming Cohen's d = 0.50). For the alpha level, a Bonferroni adjustment (5% / 2 = 2.5%) has been applied due to multiple testing with two primary endpoints. The achievable number of cases has been taken as *N* = 17 per cluster, with an assumed dropout at t1 of 10% and again 10% at t2. Conservatively, this leaves *N* = 13 cases per cluster with complete data.

Due to clustering by department, calculation of the design effect is required. For the intra-cluster correlation, a value of 0.01 has been assumed, in keeping with Adams et al. [80]. For stepped-wedge designs, power analysis is complex because, in addition to the cluster structure, the temporal effect due to the delayed start of intervention of the clusters will have to be taken into account. However, it is difficult to formulate assumptions about this temporal effect. Since we can assume a medium intra-cluster correlation, the power of the stepped-wedge design is comparable to the power of an ordinary parallel cluster randomized trial [81]. The resulting test power for testing of mean differences under the above conditions with regards to effect size, alpha level and intra-cluster correlation, has been calculated using the program nQuery. The calculation also takes into account that this is an incomplete design, since one of the clusters will be present in the intervention phase from the beginning. A total of six clusters with *N* = 13 per cluster has been assumed. This results in a test power of 94.8% (for a single endpoint), so that the power is above 80% even when multiple testing with two endpoints is taken into account.

Data will be analyzed using IBM SPSS Statistics for windows [82], MPlus [83] and Stata [84]. The content-analytical evaluation of the audio recording of the transcribed focus groups will be based on the procedure of Mayring [85].

### Ethical considerations and safety

The ReduRisk-study will be carried out without any commercial interest of the scientists, clinical staff or study participants involved. We will communicate the study content transparently for participants, the staff involved and the scientific community. The study has received a positive vote by the ethics committee of the University of Freiburg Medical Center (21–1240) and is registered with the German Clinical Trials Register (DRKS00025594, date of registration: 09/08/2021). The participants in the control group will be treated in accordance with the medical, ethical and legal standards established in Germany and at university hospitals. Participants in the intervention group will receive non-invasive, low-risk care without any restrictions to usual care. All interventions will be carried out in accordance with the hygiene and infection control regulations of the University of Freiburg Medical Center. Through the involvement of experienced health professionals as study staff, including physicians, nurses and physiotherapists, we expect study participants to be well-engaged.

### Data management

#### Data protection

As part of the preparation for the project, a cohesive data protection concept has been developed to ensure the safety of participants. The concept describes the pseudonymization process for interventionists, tablet usage and retrieval of claims data provided by the AOK. This further supports the safety of data exchange between all stakeholders. Data collection will only be performed if informed consent has been obtained.

Data collection will be done using REDCap (https://projectredcap.org/), which provides an online solution for data gathering in studies. REDCap ensures that only stakeholders with legal permission can access online personalized data. The data analysis team, for instance, has no such access rights.

All data regulation processes have been developed in conjunction with the data protection officer at the University of Freiburg Medical Center.

#### Quality control of data

Extensively documented process regulations are available, which specify quality criteria for data management and data evaluation, thus regulating quality assurance. Quality control will be done on a regular basis throughout the project.

### Public dissemination, transfer and implementation

Both positive and negative study results will be published in full, preferably in open access journals with a high impact factor and international standards in accordance with the reporting guidelines of the EQUATOR network. All relevant protocol changes will be submitted to the responsible ethics committee and will be reported when the study results are published. The publication of a user friendly manual is also planned.

### Trial status

Enrolment for the trial began in October 2021. Recruitment and data collection will continue until February 2023.

## Discussion

Multimorbidity is common in older patients. However, single-disease guidelines are still the norm for the prevention and treatment of frailty [86, 87], depression [88], type II diabetes mellitus [89] and dementia [90]. The challenges of applying such single-disease guidelines to multimorbidities is evident in their often contradictory therapeutic recommendations, with high temporal and physical overload due to accumulating treatments [58]. In 2016, the World Health Organization (WHO) first published recommendations for dealing with multimorbidity [91], subsequently followed in 2017 with guidelines for multimorbidity published in Great Britain [92] and in Germany [93]. Due to their complexity, these guidelines focused primarily on dealing with multimorbidity in general, without specifically addressing certain combinations or interactions of multiple diseases or treatments. Guidelines for appropriate deprescribing, with the feasibility of prevention and treatment of older patients with specific multiple, interactive diseases, remains elusive.

For these reasons, it is even more important to develop specific multidimensional interventions and to examine them for their effectiveness in older patients. To our knowledge, this study is the first to combine a variety of indication-specific, preventative interventions in a need- and risk-adjusted manner. This could enable their accessibility to a heterogeneous target group, whilst also examining their effectiveness through a large-scale cluster-randomized controlled study.

Since the target group is deliberately broad, it covers the entire spectrum of preventative and curative therapies in older patients, that could be employed in their usual care. We consider it ethically, scientifically and economically justifiable to exclude palliative patients from this preventative study. Accordingly, our results cannot be transferred to patients receiving palliative care or end-of-life patients.

Positive study results would support the efforts to improve multicomponent prevention and treatment in older patients. Subsequently, the ReduRisk-intervention could be integrated into usual care through multiplier training. To support such an implementation, a user-friendly manual on the ReduRisk-intervention is currently in development.

## Data Availability

Not applicable.

## Abbreviations

3D-CAM: 3-Minute Diagnostic interview for Confusion Assessment Method-defined delirium
AOK: Allgemeine Ortskrankenkasse, statutory health insurance
ADL: Activities of Daily Living
AWMF: Arbeitsgemeinschaft der Wissenschaftlichen Medizinischen Fachgesellschaften, the Association of the Scientific Medical Societies in Germany
ÄZQ: Ärztliches Zentrum für Qualität, German Agency for Quality in Medicine
BMBF: German Federal Ministry of Education and Research
CONSORT: Consolidated Standards of Reporting Trials
CRF: Case Report Form
DFG: German Research Foundation
DOS: Delirium Observation Screening
DRKS: German Clinical Trials Register
EQUATOR-Network: Enhancing the Quality and Transparency of Health Research Network
IfA: Institut für Allgemeinmedizin, Institute of Primary Care, University of Freiburg Medical Center
ISAR: Identification of Seniors at Risk screening tool
IQWIG: Institut für Qualität und Wirtschaftlichkeit im Gesundheitswesen, German independent Institute for Quality and Efficiency in Health Care
LoChro: Local, collaborative, stepped and personalized care management for older people with chronic diseases
MedQoL: Medication and Quality of Life in Frail Older Persons
MMSE: Mini-Mental-State-Examination
MOCA: Montreal Cognitive Assessment
PIM: Potentially inappropriate medication
PPO: Potential prescribing omissions
PREPARE: Prepare for Your Care
RCT: Randomised Controlled Trial
ReduRisk: Reduction of care-relevant risks for older patients during and after acute hospital care;
SEVERA: Sektion Versorgungsforschung und Rehabilitationsforschung, Section of Health Care Research and Rehabilitation Research, University of Freiburg Medical Center
SOP: Standard Operating Procedures
SPPB: Short Physical Performance Battery
UKF-Delirium Standard: Internal Standard for Delirium Prevention and Treatment of the Medical Center: University of Freiburg
UTN: Universal Trial Number
WHO: World Health Organization
ZGGF: Zentrum für Geriatrie und Gerontologie Freiburg, Center for Geriatric Medicine und Gerontology, University of Freiburg Medical Center
