# Correction: Reduction of care-relevant risks to older patients during and after acute hospital care (ReduRisk) – study protocol of a cluster randomized efficacy trial in a stepped wedge design

**DOI:** 10.1186/s12877-022-03580-9

**Published:** 2023-01-12

**Authors:** Anne Göhner, Elena Dreher, Felix Kentischer, Christoph Maurer, Erik Farin-Glattacker, Rieka von der Warth, Boris A. Brühmann, Andy Maun, Vitalii Minin, Claudia Salm, Alexander Ritzi, Gwendolyn Engelhardt, Mario Sofroniou, Sebastian Voigt-Radloff

**Affiliations:** 1 Center for Geriatric Medicine and Gerontology Freiburg, Faculty of Medicine and Medical Center - University of Freiburg, Lehenerstrase 88, 79106 Freiburg, Germany; 2 Section of Health Care Research and Rehabilitation Research, Institute of Medical Biometry and Statistics, Faculty of Medicine and Medical Center - University of Freiburg, Hugstetterstraße 49, 79106 Freiburg, Germany; 3 Institute of General Practice/Family Medicine, Faculty of Medicine and Medical Center - University of Freiburg, Elsässerstraße 2n, 79110 Freiburg, Germany

**Correction: BMC Geriatrics 22, 754 (2022)**

**https://doi.org/10.1186/s12877-022-03442-4**

After publication of this article [1], the authors reported that (1) the author name Erik Farin-Glattacker was incorrectly written as Erick Farin-Glattacker; (2) the author name Rieka von der Warth was incorrectly written as Rieka von der Wart; (3) the author Gwendolyn Engelhardt was missing.

The original article [1] has been corrected.
